# The T cell response to SARS-CoV-2: kinetic and quantitative aspects and the case for their protective role

**DOI:** 10.1093/oxfimm/iqab006

**Published:** 2021-02-23

**Authors:** Antonio Bertoletti, Anthony T Tan, Nina Le Bert

**Affiliations:** 1 Programme in Emerging Infectious Diseases, Duke-NUS Medical School, Singapore; 2 Singapore Immunology Network, A*STAR, Singapore

## Abstract

SARS-CoV-2, the etiological agent of COVID-19, triggers an adaptive immunity in the infected host that results in the production of virus-specific antibodies and T cells.

While kinetic and quantitative aspects of antibodies have been analyzed in large patient cohorts, similar information about SARS-CoV-2-specific T cells are scarce. We summarize in this review the available knowledge of quantitative and temporal features of the SARS-CoV-2 T cell response. Currently, most of the data derived only from the analysis of the circulatory compartment. Despite this limitation, early appearance, multi-specificity and functionality of SARS-CoV-2-specific T cells are associated with accelerated viral clearance and with protection from severe COVID-19.

https://mc.manuscriptcentral.com/oxfimm

T and B cells act together to resolve viral infections but are performing non-redundant functions: B cells produce antibodies that recognize directly viral proteins and can prevent virus infection of the targeted cells. T cells instead recognize viral proteins not directly but only in association with MHC-class I and MHC-class II molecules displayed on the surface of the cells. MHC-class I molecules are displayed in variable quantities at the surface of all nucleated cells of the body, and they present viral proteins that are synthesized within the cells. Consequently, T cells recognizing MHC-class I molecules complexed with viral peptides (CD8+ cytotoxic T cells) target specifically the cells where viruses are replicating. Through direct lysis of virus-infected cells or through the secretion of antiviral cytokines (mainly IFN-gamma) CD8 T cells are involved in the direct suppression of viral production and in the containment of infection. The T cells recognizing viral proteins associated with MHC-class II (CD4+ helper T cells) perform a different task. MHC-class II is expressed principally only by professional antigen presenting cells (dendritic cells, monocytes, macrophages), which are not infected but present viral antigen that has been taken up from the surrounding environment. T helper cells (or CD4 T cells), by recognizing viral antigen presented by the professional antigen presenting cells, produce diverse cytokines (IL-2, IL-21, IFN-gamma, TNF-alpha) essentially supporting the expansion and the maturation of CD8 T cells and B cells.

Despite the importance of such coordinated involvement of T and B cells in prevention and clearance of viral infections, we know that different viruses can preferentially require diverse contributions of the distinct components of adaptive immunity. For example, protection from highly direct cytopathic viruses requires a particularly efficient antibody response that limits infection of target cells. In contrast, low or non-directly cytopathic viruses are better controlled by cytotoxic T cells [1].

Such distinction is still not established in human Coronavirus infections but data in animal models support the role of T cells in viral protection. For example, mice are protected from mouse adapted SARS-CoV infection by CD4 T cells and by production of IFN-gamma [2]. In a similar model, it was also shown that clearance of SARS-CoV infection was dependent on the expansion of an early and robust virus-specific CD8 T cell response [3]. More importantly, protective studies performed in rhesus macaques challenged with SARS-CoV-2 demonstrated that CD8 T cells play an essential role in the control of infection in animals who have sub-optimal levels of neutralizing antibodies [4]. Furthermore, prospective studies in humans are starting to reveal the importance of SARS-CoV-2 T cells in disease protection [5].

In this review we will discuss, based on the knowledge currently available, quantitative and kinetic parameters of SARS-CoV-2 T cells. Other recent reviews and commentaries have already discussed aspects of SARS-CoV-2 T cells related to protection, phenotype and knowledge-gap [6-8]. We hope that a summary of the quantitative and kinetic aspects of the SARS-CoV-2-specific cellular immune response might provide a solid reference for future study design to better define the role of SARS-CoV-2 T cells in human infection and vaccination.

**Characterization of SARS-CoV-2 T cells in humans: the limitations.**

The first studies of immune cellular parameters in patients with COVID-19 reported marked lymphopenia [9,10], signs of T cell activation and cytokine production impairment on total T cells [11-13]. These initial studies investigating the activation levels and functional profile of total T cells were then followed by the characterization of the real players, T cells specific for the different proteins of SARS-CoV-2. Within a few months of the start of the pandemic, several groups showed that individuals infected by SARS-CoV-2 do not only produce antibodies but possess SARS-CoV-2-specific CD4 and CD8 T cells. The first studies were done in COVID-19 convalescents [14-22] immediately followed by characterization of individuals after asymptomatic infection [23]. Importantly, most of these studies reported also the presence of SARS-CoV-2-specific T cells in variable frequencies in unexposed healthy individuals [14-17,24].

Before discussing the kinetic and quantitative aspects of SARS-CoV-2-specific T cells we need to point out some inherent limitations of these studies in humans. The first one is that SARS-CoV-2 T cells in humans have so far only been analyzed in peripheral blood but studies in animal models showed that virus-specific T cells are preferentially recruited into the respiratory tract [2,3].

Circulating T cells detected in the blood therefore are a fraction of the total pool of SARS-CoV-2 T cells present in an infected patient. The numerical value of such https://mc.manuscriptcentral.com/oxfimm fraction is unknown: it is unlikely to be a fixed ratio but it will be dependent on the degree of inflammatory processes present in the respiratory tract. Since the surface area of the respiratory tract is large (about 145 $m^2$), it is likely that a very large quantity of T cells is trapped there during infection, particularly in patients with severe disease. This could also explain the marked general lymphopenia detected in their peripheral blood [9,22]. Of note, in severe influenza a specific enrichment of influenza-specific T cells in the lung (about 45 times more than blood) was reported [25]. Furthermore, functional differences between the virus-specific T cells resident in different anatomical sites were also reported in animal studies [2]. CD4 T cells present in the airway produce more cytokines than other parenchymal or vascular T cells and their ability to produce concomitantly IFN-gamma and IL-10 appears to be required for optimal protection [2].

Limitations are also introduced by the methods utilized for T cell analysis.
T cell analysis with MHC-class I or class II multimers are precise and do not require the functionality of the T cells, but a more limited T cell repertoire is detectable in comparison to the use of an unbiased peptide library. For example, SARS-CoV-2-specific T cell analysis performed with a large library of MHC-class I multimers detected some SARS-CoV-2-specific CD8 T cells, but robust frequencies were detected only after in vitro enrichment [26,27]. Direct visualization of T cells with MHC-tetramers allows also to study their possible “functional phenotype” like the expression of exhaustion/inhibitory markers (PD-1, TIM-3, CTLA-4). However, the significance of the expression of these markers during acute viral infection correlates more with the activation status than with functional exhaustion [28].

The number of epitopes visualized in individual patients by MHC-tetramers complexed with peptides selected by epitope predictive algorithms is low (2-3 epitopes) [26] in comparison to what was detected with assays based on the use of peptide libraries (10-15 different pools positive) [14,20] or by minigenes expressing viral antigens [29] (~3 epitopes for each MHC-class I). However, for their detection, T cells must be functionally efficient and produce cytokines or proliferate or express activation markers and there is accumulating evidence showing a degree of reduced production of cytokines (particularly IFN-gamma) in SARS-CoV-2 T cells of symptomatic COVID-19

https://mc.manuscriptcentral.com/oxfimm patients [19,30]. The data are still preliminary, and a full functional T cell exhaustion of SARS-CoV-2 T cells has not been demonstrated [28]. Nevertheless, T cells of symptomatic patients secrete lower levels of IFN-gamma than the ones of individuals with asymptomatic infection [30]. This could be one of the reasons why analysis of T cells with “activation induced markers (AIM)”, namely CD40L, OX40 and CD69 detect more SARS-CoV-2 T cells than methods based on IFN-gamma production (intracellular cytokine staining and ELISpot). One the other hand, it is important to mention that detection of SARS-CoV-2-specific T cells utilizing AIM expression obtained after 24 hours of peptide stimulation has the potential to induce bystander T cell activation which might inflate the number of T cells detected.

Which specific functional assays might provide T cell quantifications that correlate more with their potential protective ability is still unknown, but certainly worth future evaluation. In HIV infection for example, it is only the number of T cells producing high levels of cytokines that correlates with viral protection [31].

In addition, performing a comprehensive analysis of the full repertoire of T cells against the whole SARS-CoV-2 proteome is challenging. The full proteome of SARS-CoV-2 is large, about 10.000 AA. Antibodies have protective ability when targeting the antigen located on the surface of the virus and particularly the region binding to the ACE2 receptors [32,33]. In contrast, understanding the protective ability of different T cells is complex and not necessarily correlated with their quantity: T cells can be specific for all the distinct viral proteins produced within an infected cell. The endogenous synthesized viral proteins are processed and presented as epitopes which can elicit protective T cells irrespective to their quantitative expression [34,35]. Furthermore, only few studies with a limited number of COVID-19 recovered patients have performed a comprehensive analysis of virus-specific T cells to the full SARS-CoV-2 proteome with peptide libraries [14,20] or with minigenes expressing antigens [29].

A more robust set of data is instead available for T cells recognizing structural proteins, particularly the viral antigens spike, membrane and nucleoprotein. These proteins represent less than a third of the whole SARS-CoV-2 proteome [36,37]. Even though they might not always contain the immunodominant epitope [29], they are immunogenic for both CD4 and CD8 T cells, and appear to elicit a strong T cell https://mc.manuscriptcentral.com/oxfimm response both in symptomatic and asymptomatic individuals [14,19,20,23,30,38]. There are however also criticisms of investigating T cells utilizing only synthetic peptides. T cells activated by peptides presented by antigen presenting cells expressing high quantity of MHC-class I or class II molecules might just be "peptide specific" or low affinity T cells and not really able to recognize virus infected targets. This might overestimate the number of T cells able to recognize efficiently virus infected cells and this criticism has been raised particularly for the significance of SARS-CoV-2 T cells present in healthy unexposed individuals [39].

**Kinetics of SARS-CoV-2 T cells: Induction and expansion.**

Despite all the different caveats, the results produced by many different groups are starting to define some clear pattern of induction, expansion and contraction of the humoral and cellular immune response during and following SARS-CoV-2 infection.

The kinetics profile of the antibody response after coronavirus infection is well defined. Several studies have shown that antibodies are detectable in the sera of the majority of SARS-CoV-2 infected symptomatic individuals within 4-5 days after the onset of symptoms with levels that rise for at least two weeks and that are higher in cases with more severe disease [40-43].

The knowledge of virus-specific T cell kinetics and its association with disease severity is instead still limited since fewer works have examined the kinetics of SARS-CoV-2-specfic T cells during the acute phase of infection.

We know that SARS-CoV-2 T cells are detected, like antibodies [44], in almost the totality of infected individuals after recovery. Virtually all SARS-CoV-2 infected symptomatic individuals positive for antibodies against NP or Spike possess a broad repertoire of T cells recognizing different structural proteins of SARS-CoV-2 (i.e.NP, M, Spike, ORF3a) [14,19,20].

Importantly, there is however also a discordance between virus specific antibody levels and T cell responses. For example, in some individuals, particularly the ones with mild or asymptomatic infection, SARS-CoV-2 multi-specific T cells are detectable despite the absence of a concomitant antibody response [23,30,45]; furthermore, different levels of neutralizing antibodies are detected in convalescent COVID-19 patients with similar quantity of SARS-CoV-2 T cells [46].

https://mc.manuscriptcentral.com/oxfimm

Like antibodies, SARS-CoV-2 T cells are detected within a week from onset of symptoms (likely about 7-10 days from infection - time of infection cannot be precisely determined in human). This has been clearly shown in symptomatic COVID-19 patients [19,47] and such T cells can be detected remarkably early. For example, Schulien et al. visualized SARS-CoV-2 CD8+ T cells directly with MHC-multimers at day 1 after symptoms, and showed that these virus-specific CD8 T cells were already activated [27]. In other studies, where T cells were detected with functional assays (ELISpot for IFN-gamma producing T cells or AIM), functionally responsive SARS-CoV-2 T cells were detected at 3-5 days after onset of symptoms (Figure1) [19,47]. This accelerated induction of SARS-CoV-2 T cells indicates that in many infected individuals, a perfect rapid and coordinated activation of innate and adaptive immunity is occurring. Note that for example, infections with viruses which evade or suppress innate immune recognition, like HBV or HCV, trigger an induction of virus-specific T cells only after 4-6 weeks from infection [48]. Indeed, the rapid expansion of the adaptive immune response is a proxy for the efficiency of the innate immunity triggering.

The early induction of virus-specific antibodies and T cells after SARS-CoV-2 infections are however associated with different outcome.

While antibodies against Spike and NP can be detected early in all patients irrespective of clinical outcome, the early detection (<10 days from symptoms onset) of SARS-CoV-2 T cells in COVID-19 patients is associated with milder disease course and accelerated viral clearance [19,47]. Delayed appearance (>15 days from symptoms onset) and weak induction of SARS-CoV-2 T cells was observed only in patients with severe COVID-19 [15,19,47] further supporting the concept that SARS-CoV-2 T cells are indispensable for viral control and that antibodies alone cannot clear an established infection. In severe COVID-19, the defective induction of a fully functional virus-specific T cell response is then associated with what has been defined as "immunology misfiring" [13]: robust and persistent activity of different components of innate immunity with a lack of a clear induction of a Th1-like immune response.

An open question about the SARS-CoV-2 T cell kinetics is whether a rapid virus-specific T cell induction occurs also in asymptomatic infection. The lack of symptoms conceals the possibility to recruit asymptomatic individuals at the beginning of infection and the lower level of SARS-CoV-2 antibodies detected in these individuals [42] have suggested that virus-specific B and T cell levels are directly proportional to symptom level. Initial data derived from asymptomatic SARS-CoV-2 exposed individuals months after recovery supported this interpretation [20,45]. However, a robust induction and functionally efficient SARS-CoV-2 T cell response to levels even superior to what is detectable in symptomatic COVID-19 patients has been found in recently infected asymptomatic individuals [23,30]. We think it is plausible to hypothesize that asymptomatic infection is characterized by a very rapid and efficient induction of a virus-specific cellular immune response.

One other question related to SARS-CoV-2 T cell induction is whether T cells specific for the different SARS-CoV-2 viral proteins are induced with different kinetics and whether a preferential induction of a specific T cell determinant is associated with better or worse viral control. For antibody responses, preferential induction of spike-specific antibodies is associated with faster SARS-CoV-2 clearance and mild disease while an augmented anti-NP-specific antibody response is associated with worse diseases outcome [49]. However, if such findings are explained by the different protective efficiency of the antibodies against NP or Spike, a hypothetical parallel for T cells cannot be derived since we don't have any indications if T cells specific for different proteins have distinct protective values.

At the moment, most of the data indicate that the first detectable SARS-CoV-2-specific T cell response is already multi-specific, with T cells simultaneously recognizing different epitopes in different proteins already at early stages of infection [15,19,50]. However, in a small study of patients analyzed longitudinally during the early phase of infection we noted a dynamic modulation of the hierarchy of SARS-CoV-2-specific T cells. T cells specific for ORF7 and ORF8 regions of SARS-CoV-2 were induced early and were more robustly detected in the early phases of infection than in convalescence [47]. These data need to be confirmed in larger studies, but the possibility that some viral antigens can be more efficient than others in triggering a specific T cell response might occur and it is compatible with different possibilities.

T cells specific for non-structural proteins, like for example ORF-1 coded proteins, might be induced earlier as a consequence of the complex replication strategy of Coronaviruses that requires the formation of a viral replicase-transcriptase complex https://mc.manuscriptcentral.com/oxfimm essential for the subsequent transcription of the viral genome [51]. The early produced non-structural proteins might trigger T cells ahead of T cells specific for structural viral proteins. Indications that this might occur are starting to appear in the literature, with demonstration of dominant epitopes detected in the ORF-1 region [29]. One other possible explanation for the differential kinetics of induction of T cells with different specificities can be derived from the pre-existence of SARS-CoV-2 cross-reactive T cells prior to the infection induced by other coronaviruses, vaccinations or other pathogens. As we briefly mentioned earlier, the existence of T cells specific for SARS-CoV-2 peptides in unexposed individuals has been reported in multiple studies [14-17,24] and their impact in the protection and pathogenesis of SARS-CoV-2 infection is highly controversial [39,52,53].

Nevertheless, the possibility that SARS-CoV-2 T cell induction can be augmented by pre-existent memory T cells induced by other coronaviruses has been recently reported [54].

Furthermore, the first SARS-CoV-2 vaccines are designed to induce spike-specific immunity only, since spike antibodies have protective ability. Spike-specific T cells should theoretically have identical protective values than T cells specific for other viral antigens. It will be however interesting to evaluate the impact that changes in the T cell immunodominance induced by vaccination will exert on the protective ability of the multi-specific T cell response induced physiologically by the natural infection.

**Kinetics of SARS-CoV-2 T cells: contraction phase.**

After the expansion phase, that usually lasts for 10-20 days after symptom onset, T cells slowly decline but remain still detectable in the majority of individuals tested at least within 6-8 months after infection [30,45,54-58].

The kinetics of contraction of CD8 and CD4 T cells appears to slightly differ: both SARS-CoV-2-specific CD4 and CD8 T cells are present within the first 2 weeks after onset of symptoms, but while CD8 T cells show signs of progressive reduction after viral clearance (>1 month after infection) [19,29], the SARS-CoV-2 CD4 T cell frequency is more stable and appears higher in individuals tested in the initial recovery phase (1-2 months after infection) than immediately after infection [19]. These differential kinetics of expansion and contraction can explain the preferential detection https://mc.manuscriptcentral.com/oxfimm of CD4 helper T cells in studies that have been investigated SARS-CoV-2 convalescent individuals [14,16,20,54-58]and it mirrors the recent evidence of an identical persistence and progressive increase of SARS-CoV-2-specific B cells during the convalescent phase [56,59]. The causes of such phenomena have not been demonstrated, but a sound hypothesis is that viral antigens can persist as an antigenic depot in dendritic cells within lymph nodes [59].

Indeed, initial reports detecting a decline of SARS-CoV-2 antibodies immediately after the acute phase of infection [40,60] have suggested that SARS-CoV-2-specific T cells were able to persist longer than the corresponding antibody response. However, more extended cross-sectional data showed that the virus-specific humoral and cellular immunity are, after an initial contraction phase [40,47,60], remarkably stable for at least 6-8 months after infection [30,56,57]. A recent cross-sectional study of COVID-19 recovered individuals analyzed more than 6 months after infection has calculated t1/2 of approximately 3.5 months for CD4 and CD8 T cells detected with activation induced markers [56]. One other study that analyzed T cells with ELISpots has shown that 6 months after recovery, all COVID-19 convalescents have detectable T cells at variable frequency with a mean of 250 spots x million cells for T cells specific for NP, membrane and Spike [55].

One other unknown related to the contraction phase of SARS-CoV-2 T cells is whether this is faster in SARS-CoV-2 infected individuals in relation to the severity of disease. A recent comparative analysis of the SARS-CoV-2 specific T cells in asymptomatic versus symptomatic groups detected a very similar magnitude of SARS-CoV-2 T cell responses within 1-3 months after infection [30]. However, a faster decline of the SARS-CoV-2 T cell quantities was observed in asymptomatic individuals studied 6 months after infection compared to symptomatic COVID-19 patients at the same timepoint [55]. More studies will be needed to define whether cellular immunity decline is associated with the degree of symptom severity.

In conclusion, in these initial paragraphs we summarized most of the presently available data related to the kinetics of appearance and disappearance of SARS-CoV-2 T cells. Experimental evidence indicates that the kinetics of induction of a multi-specific T cell response is an important parameter of antiviral efficacy and that such multi-specific T cells can persist after viral clearance. Whether the quantity of T cells persisting as memory cells months after recovery will be able to mediate protection is still unknown as it is unknown whether those kinetic parameters of T cells are associated with age, sex and concomitant pathologies, variables that play important roles in the severity of SARS-CoV-2 infection.

**Quantity and immunodominance of the SARS-CoV-2 T cell response.**

What is the overall magnitude of virus specific T cells present in SARS-CoV-2 infected patients? The quantity of virus-specific T cells in other viral diseases varies: some viruses (like HCMV, EBV, HIV) elicit high quantities of T cells which can often reach frequencies of 5-10% of total circulating T cells [31,61,62], while other virus-specific T cells (HBV, HCV) are seldom detected in blood at frequencies above 0.1% [63]. These differences are not due to a single cause, but the fact that different viruses target different cell types and organs is an important factor. T cells are not evenly distributed among the body. As we have already mentioned, T cells in SARS-CoV-2 infected individuals have been so far analyzed only in peripheral blood but studies in animal models [2,3] or other human respiratory diseases [25] show that virus-specific T cells can be preferentially recruited in the respiratory tract.

We try to summarize in Table 1 the known frequencies of total circulating SARS-CoV-2-specific T cells detected in SARS-CoV-2 infected individuals published in different manuscripts. Note that all T cells responses that have been so far reported in SARS-CoV-2 infection are Th1/Th0-type and not Th2-type. We also compared the frequency of Spike-specific T cells induced by natural infection with the one induced by the SARS-CoV-2 vaccines that have terminated Phase II clinical trials at the time of writing and in which Spike-specific T cell quantities were reported [64-71]. Frequency of T cells found within 1-3 months from infection or 2-4 weeks after vaccination are displayed (Table1).

Overall, the results so far accumulated show that SARS-CoV-2 T cell quantity is, like antibodies, highly heterogeneous among different patients. On average T cells specific for the different structural proteins (NP, M and Spike) within 2-3 months from COVID-19 recovery are present at 0.1-1% of total CD4 or CD8 T cells with methods that detect T cell activation induced markers (AIM). ELISpot results show quantities of around

100-1000 spots x million PBMC against different individual proteins. The overall quantity does not differ between mild symptomatic and asymptomatic SARS-CoV-2 T exposed individuals, but the functionality of SARS-CoV-2 T cells in asymptomatic seems superior [30]. These data are in accordance also with results obtained in patients with severe COVID-19 who display lower quantities and lower functionality of SARS-CoV-2-specific T cells than patients with mild disease [19,38].

The frequency of Spike-specific T cells induced by different preparations of vaccines is also reported. The different vaccine preparations, perhaps with the exception of the inactivated Sinovac vaccine [68], trigger variable quantities of Spike-specific CD4 and CD8 T cells. Note however that a quantitative comparison between Spike-specific T cells induced by natural infection and vaccination is often incongruous since Spike-specific T cells were measured latest at 28 days after vaccination [70].

It remains to be analyzed whether vaccinations can induce a long-lasting memory T cell response to levels similar to what can be achieved by natural infection and whether the induced Spike-specific T cells play a role in the vaccine efficacy.

There are two other important questions related to the magnitude of SARS-CoV-2 T cells: one is its relation with antibody responses and the second is related to the immunodominance of different epitopes.

The relation between the magnitude of SARS-CoV-2 T cells and antibodies is “intricate”. The quantity of neutralizing antibodies and total SARS-CoV-2-specific CD4 and CD8 T cells correlates in convalescent mild COVID-19 patients [19,55]. Also, the frequency of Spike-specific CD4+CXCR5+ (a phenotype marker that defines circulating follicular helper T cells) was positively correlated with high level of Spike antibody and neutralizing activity [21], despite most of the T cell response is targeting the Spike protein outside the RBD region [50]. However, neutralizing antibody levels were inversely correlated with the quantity of Spike-specific CD4+ CCR6+ CXCR3- T cells able to produce IL-17 [21] and no correlations were also detected between NP-specific T cell responses (Th1-like) and the magnitude and titers of neutralizing or NP-specific antibodies [16].

Furthermore, even though a coordinated expansion of both cellular and humoral arms of adaptive immunity is often found in mild but not severe COVID-19 patients [19], a robust SARS-CoV-2 T cell response in the absence of antibodies was found in some patients with mild COVID-19 who successfully control the infection [19,23,30,45,54].

https://mc.manuscriptcentral.com/oxfimm

Interestingly, the scientific literature does not report any evidence of COVID-19 patients who controlled the infection only by eliciting an antibody response in the absence of detectable SARS-CoV-2 T cells [19,47], while high titers of antibodies (even neutralizing antibodies) are present in severe disease [72].
The discrepancy between presence of virus-specific T cells and absence of specific antibodies is detected also in some asymptomatic cases [23,30] and in other coronaviruses infections like MERS, in whom subjects who were likely in contact with the viruses present MERS-specific T cells but no antibodies [73,74]. Similarly, SARS-CoV-specific T cells (CD4 and CD8 T cells) can be detected 11 and 17 years after SARS-CoV infection [16,75] in the absence of detectable antibodies.
The cause of this detection discrepancy is still unknown. Some individuals can mount initially exclusively a cellular or a humoral response after exposure. Demonstration of extrafollicular B cell maturation particularly in severe COVID-19 patients [76] might partially explain the presence of strong antibody responses in the absence of T cells [77,78] but the immunological mechanisms that lead to the exclusive induction of cellular immunity are not known. An alternative interpretation of these results is that humoral and cellular responses might decay in some people with different kinetics. The data collected within the first 6 months after infection do not support such interpretation since waning of antibody titers and T cells appear similar [55], but it is still possible, as seen in SARS-CoV infection [79], that at later time points the rate of reduction of antibodies and T cells could diverge.

Quantitative features of the antiviral T cell response are not only related to the total numbers of SARS-CoV-2 T cells present but also on their diversity. CD8 and CD4 T cells can recognize different epitopes located in the same and/or different proteins. The SARS-CoV-2 T cell response in COVID-19 recovered individuals is highly multi-specific with T cells recognizing not only multiple proteins [14,20,23], but also multiple regions within a single protein [16,26,29,50]. This diffuse repertoire of T cells recognizing several epitopes displays a very heterogeneous TCR repertoire with the presence of some public TCR usage shared by some individuals [80].

Whether multi-specificity is the key of protection is still uncertain. A correlation between multi-specificity and mild disease has been reported [26]. Hypothetically, the ability to mount a broad T cell response against several epitopes located in different viral proteins might be advantageous and might avoid the selection of mutated viruses able to escape CD8 T cell recognition. In other viral infections, (i.e.HBV) the multi-specificity of the T cell response appears to be an important determinant of viral clearance [81]. Nevertheless, the data at the moment are only showing associations and not causality. It might be possible that despite the broad T cell multi-specificity observed in SARS-CoV-2 recovered individuals, robust T cells specific for a single or limited number of epitopes present in a single protein can be equally protective. Detailed analysis of different epitopes and their element of restrictions are starting to appear in the scientific literature [26,29,50] and such information will be indispensable to understand whether single T cell determinants are more or less important for viral clearance during natural infection. This information will have also to be translated into the analysis of T cells induced by vaccines that, currently, include only the single Spike protein or only its RBD region. Such vaccines can certainly not induce the broad T cell repertoire as seen by the natural infection, and this Spike-focused T cell induction might not be ideal. Nevertheless, the protective response induced by a vaccine is likely to be mediated by the ability to obtain a coordinated induction of both Spike-specific antibodies and T cells. Note that a very efficacious prophylactic vaccine, the one against HBV, also elicits a combined antibody and T cell response focused only against the S antigen of the virus (the envelope protein of HBV) [82] despite HBV control after natural infection is correlating with induction of a multi-specific T cell response [81]. The two viruses display different viral replication kinetics (peak viral replication for SARS-CoV-2 within 1-2 week from infection [72], 4-6 weeks after infection for HBV [48]), but this comparative hypothesis is in line with the initial evidence of the protective efficacy of Spike-based vaccines recently reported.

## Concluding remarks

The SARS-CoV-2 pandemic has changed our life. It has not only caused the loss of many people but it has also compromised and changed social and economic habits. The response to this crisis has also transformed the scientific world. The pace of new scientific information related to SARS-CoV-2 has been growing exponentially. Scientific hypotheses are confirmed or become obsolete not after years, but after few months. When we accepted to write this review on virus-specific T cells in SARS-CoV-2 infection, we did not fully realize that this time we were asked to summarize a scientific argument that is far from being established but is in dynamic development. The risk to describe phenomena that could soon be considered obsolete or insignificant is high. Nevertheless, we report here kinetics and quantitative features of virus-specific T cells that in our opinion have fundamental importance to define their role either in the protection or in the pathogenesis of the disease caused by SARS-CoV-2. We think that the majority of the data support a protective role of the quantity and diversity of virus-specific T cells in SARS-CoV-2 infection: T cells are indispensable in mouse models of coronavirus infection, are critical in protective studies in rhesus monkeys and their early presence, functionality and multi-specificity is correlated with protection and rapid viral control in infected individuals. Nevertheless, more data are needed to fully understand the role of virus-specific T cells in different groups of patients with different disease severity, the relation of SARS-CoV-2 T cell quantity and function with age, sex and other pathologies affecting infected individuals, or the role of T cells in protective vaccination and in the pathogenesis of long-term COVID-19, in which a possible pathogenetic role of T cells can certainly not be excluded.

https://mc.manuscriptcentral.com/oxfimm

**Acknowledgements**

This study is supported by the Singapore Ministry of Health's National Medical Research Council under its COVID-19 Research Fund (COVID19RF3-0060), the Singapore Ministry of Health's National Medical Research Council MOH-000019 (MOH-StaR17Nov-0001), and National Research Foundation, Singapore (NRF-CRP17-2017-06).

**Data Availability Statement**

Data analyzed in this study were a re-analysis of existing data, which are openly available at locations cited in the reference section.

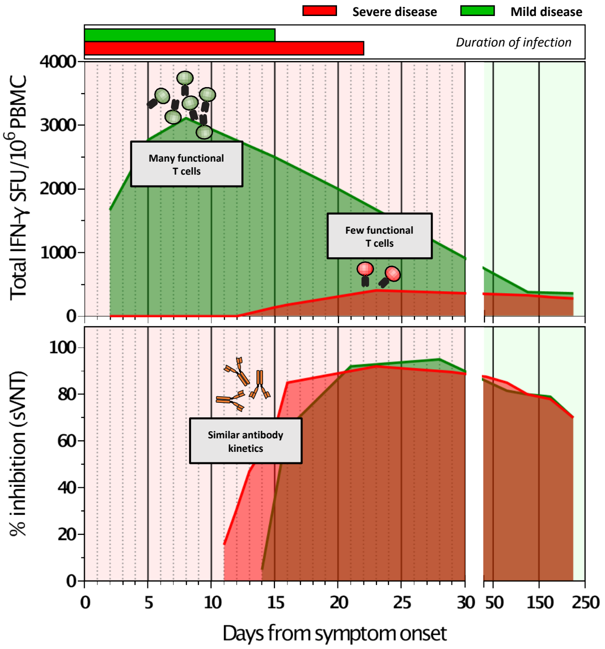

**Figure 1. Kinetics of SARS-CoV-2 specific T cell and antibody response in COVID-19 patients with mild and severe disease.**

| Study | Severity | Disease state | Sampling date (post disease onset) | Type of T cell assay | Specificity | Median SFU/mil PBMCs |
|---|---|---|---|---|---|---|
| Sekine et al - Cell | Mild | Convalescent | 1-3 months | IFN-γ ELISPOT | S | ~110 |
| | Mild | Convalescent | 1-3 months | IFN-γ ELISPOT | S,M,N | ~310 |
| | Severe | Convalescent | 1-3 months | IFN-γ ELISPOT | S | ~110 |
| | Severe | Convalescent | 1-3 months | IFN-γ ELISPOT | S,M,N | ~530 |
| Peng et al - Nat Immunol | Mild | Convalescent | 1-3 months | IFN-γ ELISPOT | S | ~250 |
| | Mild | Convalescent | 1-3 months | IFN-γ ELISPOT | S,M,N | ~500 |
| | Severe | Convalescent | 1-3 months | IFN-γ ELISPOT | S | ~500 |
| | Severe | Convalescent | 1-3 months | IFN-γ ELISPOT | S,M,N | ~1050 |
| Le Bert et al - JEM | 75% mild/25% severe | Convalescent | 1-3 months | IFN-γ ELISPOT | S | ~80 |
| | 75% mild/25% severe | Convalescent | 1-3 months | IFN-γ ELISPOT | S,M,N | ~350 |
| | 75% mild/25% severe | Asymptomatic | N.A | IFN-γ ELISPOT | S | ~60 |
| | 75% mild/25% severe | Asymptomatic | N.A | IFN-γ ELISPOT | S,M,N | ~250 |

| Study | Severity | Disease state | Sampling date (post disease onset) | Type of T cell assay | Specificity | Median+ / CD4 | Median+ / CD8 |
|---|---|---|---|---|---|---|---|
| Grifoni et al - Cell | ALL | Convalescent | 1-3 months | AIM | S | ~0.2% | ~0.1% |
| | ALL | Convalescent | 1-3 months | AIM | S,M,N | ~0.5% | ~0.25% |
| Moderbacher et al - Cell | ALL | Convalescent | 1-3 months | AIM | S | ~0.3% | ~0.03% |
| | ALL | Convalescent | 1-3 months | AIM | S,M,N | ~0.65 | ~0.1% |
| | ALL | Acute | <1 month | AIM | S | ~0.08% | ~0.02% |
| | ALL | Acute | <1 month | AIM | S,M,N | ~0.16% | ~0.07% |
| Weiskopf et al - Sci Immunol | Severe | Acute | <1 month | AIM | S | ~0.6% | ~1% |
| Breton et al - JEM | 80% mild/20% severe | Convalescent | 1-6 months | IFN-γ ICS | S,M,N,ORF3a | ~0.25% | ~0.1% |
| Dan et al - Science | 90% mild/10% severe | Acute/Convalescent | up to 8 months | AIM | S,M,N,ORF3a, NSP3 | ~1.1-0.4% | ~1-0.3% |

**Table 1A. Summary of SARS-CoV-2 specific T cell responses induced by natural infection.**

| Study | Age group | Type of T cell assay | When? | Max achievable median response (SFU per mil PBMCs; SFU per mil CD4/CD8 T cells; IFN-γ pg/ml) |
|---|---|---|---|---|
| Pfizer - mRNA | 18-55 | IFN-γ ELISPOT | 7 days post boost / 28 days post prime | ~2000 / 2000 |
| | 18-55 | IFN-γ ELISPOT | 28 days post vaccine / no boost | ~50 / 50 |
| Astrazeneca - Adenoviral | 18-55 | IFN-γ ELISPOT | 14 days post vaccine / no boost | ~800 |
| | 18-55 | IFN-γ ELISPOT | 28 days post vaccine / no boost | ~550 |
| CanSino - Adenoviral | 18-55 (10% >55) | IFN-γ ELISPOT | 28 days post vaccine / no boost | ~100 |
| Gamaleya - Adenoviral | 18-60 | IFN-γ secretion | 28 days post vaccine / no boost | ~30 pg/ml |
| Sinovac - Inactivated | 18-59 | IFN-γ ELISPOT | 14 days post boost / 28 days post prime | ~50 |
| Sinopharm - Inactivated | 18-59 ; >60 (1:1) | NOT DONE | N.A | N.A |

| Study | Age group | Type of T cell assay | When? | Max achievable median response (%IFN-γ+ out of CD4/CD8 T cells) |
|---|---|---|---|---|
| Moderna - mRNA | 18-55 | IFN-γ ICS | 14 days post boost / 42 days post prime | ≤0.2 / ≤0.1% |
| | >56 | IFN-γ ICS | 14 days post boost / 42 days post prime | ~0.3 / ≤0.1% |
| Janssen J&J - Adenoviral | 18-55 | IFN-γ ICS | 14 days post single dose | ~0.1 / 0.09% |
| | >65 | IFN-γ ICS | 14 days post single dose | ~0.3 / 0.05% |

**Table 1B. Summary of SARS-CoV-2 specific T cell response induced by different vaccines.**
